# Healthy lifestyle is inversely associated with mortality in cancer survivors: Results from the Third National Health and Nutrition Examination Survey (NHANES III)

**DOI:** 10.1371/journal.pone.0218048

**Published:** 2019-06-26

**Authors:** Nena Karavasiloglou, Giulia Pestoni, Miriam Wanner, David Faeh, Sabine Rohrmann

**Affiliations:** 1 Division of Chronic Disease Epidemiology, Institute for Epidemiology, Biostatistics and Prevention, University of Zurich, Zurich, Switzerland; 2 Cancer Registry Zurich and Zug, University Hospital Zurich, Zurich, Switzerland; 3 Health Department–Nutrition and Dietetics, Bern University of Applied Sciences, Bern, Switzerland; Nagoya University, JAPAN

## Abstract

Individual lifestyle behaviors have been associated with prolonged survival in cancer survivors, but little information is available on the association between combined lifestyle behaviors and mortality in this population. Data from 522 cancer survivors participating in the Third National Health and Nutrition Examination Survey (NHANES III) were analyzed. Behaviors pertaining to lifetime healthy body weight maintenance, physical activity, smoking, diet quality (assessed by the Healthy Eating Index) and moderate alcohol consumption were combined in a lifestyle score (range 0–5). Cox proportional hazards regression models were used to calculate multivariable-adjusted hazard ratios (HR) and 95% confidence intervals (CI). Both in continuous and categorical models, the lifestyle score was statistically significantly associated with lower mortality in the total study population (HR_continuous_ = 0.81, 95% CI: 072, 0.90, per 1 unit increase; HR_1-2 vs. 0 total_ = 0.71, 95% CI: 0.56, 0.92; HR_3-5 vs. 0 total_ = 0.57, 95% CI: 0.38, 0.85, in the fully adjusted model) and in sex-specific analyses.

Cancer survivors with high or moderate lifestyle score had lower risk of premature death compared to survivors with zero lifestyle score. Future studies are required in order to verify our findings and to investigate underlying mechanisms of the mortality-adherence association.

## Introduction

Cancer is the second leading cause of death in the United States [1]. Screening programs, early detection, and medical advancements have led to a decrease in cancer death rates [1] and an increase in survival among cancer patients [2]. However, little is known about survivors’ lifestyle behaviors and how they influence survival [3, 4]. A recent report highlighted that while there is not sufficient evidence in order to form recommendations for cancer survivors, there are indications that healthy body weight, physical activity, and dietary factors post-diagnosis may be associated with longer survival [5].

Few studies have reported an association between individual potentially modifiable lifestyle behaviors and survival in cancer patients and their results are contradicting. Higher body weight and/or obesity [6, 7], smoking [8–10] and alcohol intake [11] have been associated with increased mortality. Engaging in physical activity [7, 8, 10, 12–15], adhering to a high-quality a-priori or a-posterior defined dietary pattern [10, 16–19] and consuming some foods/food-groups [10, 20] have been associated with lower risk of death. However, a number of studies failed to detect the aforementioned associations and reported mixed findings [10, 15, 21–23].

Studies focusing only on individual lifestyle behaviors may overlook interactions between lifestyle behaviors that could potentially modify their association with mortality. Analyses combining a number of healthy lifestyle behaviors indicate that cancer survivors who adhere to healthy weight, physical activity, and diet recommendations have lower mortality compared to those who do not [4, 24, 25]. Lower all cause-mortality was also reported for survivors who only followed some of the recommendations [4].

Based on the possible association of combined healthy lifestyle behaviors with decreased mortality in cancer survivors and the limited number of studies on the topic to date, we believe that this association warrants further investigation. In our analyses, we examined the association of healthy lifestyle behaviors, expressed as a lifestyle score, with mortality in a population of cancer survivors.

## Methods

### Population

Data from the Third National Health and Nutrition Examination Survey (NHANES III) were used in the analyses. The methodology of the NHANES III has been described in detail elsewhere [26]. In short, the NHANES III was a nationwide survey, conducted between 1988 and 1994 in the United States. NHANES III participants were interviewed and underwent physical examinations in a mobile examination center. NHANES III data are publically available and can be accessed online (https://wwwn.cdc.gov).

In these analyses, only participants with a self-reported previous cancer diagnosis were included. Participants were defined as cancer survivors if they answered “yes” to the question, “Has a doctor ever told you that you had other cancer?” Participants with self-reported skin cancer diagnosis (n = 117; answered “yes” to the question, “Has a doctor ever told you that you had skin cancer?”), with missing information on the healthy lifestyle behaviors (n = 127) or any of the confounding variables (n = 14), were excluded from the analyses. The final study population included 522 participants. Due to the low count of participants per cancer type, we could not perform sub-analysis by cancer type. To account for the time between diagnosis and entry in the study we calculated the difference between the age at study entry and the self-reported age at cancer diagnosis (answer to the question “Age when 1st told had other cancer”).

### Lifestyle behaviors

Dietary information was obtained with 24-hour dietary recall interviews, using an automated data collection instrument. Data collection was scheduled as such as to include interviews all days of the week and throughout the year. To assess the diet quality, the Healthy Eating Index (HEI) score was calculated for each participant based on their dietary information. The HEI is a diet quality index developed by the U.S. Department of Agriculture that includes 10 equally weighted distinct components. Each participant’s individual score was computed by summing their score on the different components. The score ranges from 0 to 100 [27], and the higher a participant’s HEI score, the better the diet according to the Dietary Guidelines for Americans and the Food Guide Pyramid [26].

To minimize possible reverse causality resulting from weight loss due to the cancer diagnosis, treatment or other preexisting illnesses, lifetime healthy body weight maintenance was used instead of current body weight. The self-reported highest body weight attained (in pounds) over the life course and the measured height was used to estimate the lifetime highest BMI for each participant. After conversion of the highest weight to the appropriate scale, lifetime highest BMI (as a proxy to lifetime healthy body weight maintenance) was calculated as weight (in kg)/ height^2^ (in meters).

Details of the data collection for physical activity have been published elsewhere [28]. Briefly, participants were asked how frequently they performed leisure time exercise or physical activities in the past month. The duration of the physical activity was not considered in NHANES III. Answers were coded as “times per week” using the conversion factor 4.3 weeks per month. In this study, only participation in physical activities of moderate to vigorous intensity (METs 3–8) was considered. Participants were grouped according to their weekly frequency of moderate to vigorous physical activity (0, 0 to < 5 and ≥5 times/week). Information on smoking habits was collected via a self-reporting questionnaire and study participants were grouped as never, former or current smokers. Information on alcohol intake (g/day) was assessed via 24-hour dietary recall interviews.

### Lifestyle score

A score was created to reflect the number of healthy lifestyle behaviors each participant adhered to. Participants were assigned one or zero points, depending on whether or not they adhered to each healthy lifestyle behavior. One point was assigned to each of the following behaviors: Never smoker (in order to reflect lifetime increased disease risk, we did not differentiate the risk between former and current smokers), lifetime healthy body weight maintenance (expressed as lifetime highest BMI between 18.5 and 24.9 kg/m^2^; using the World Health Organization’s classification [29]), participation in moderate to vigorous physical activity 5 times or more times per week (aiming to capture those adhering to the World Health Organization’s recommendation of 150 minutes weekly activity of moderate intensity [30]), moderate alcohol consumption (5-15g per day for females and 5-30g per day for males) and high diet quality (expressed as HEI score in the highest 40% of the study population distribution (similarly to Li et al. [31]; HEI score >69.3)). The sum of the of all lifestyle behaviors was the lifestyle score for each participant. Therefore, the lifestyle score could range from 0 to 5points. Since many cancer survivors are advised to abstain from smoking and limit their alcohol consumption, we divided participants into three groups; the first group consisted of participants with lifestyle score 0, the second consisted of participants with lifestyle score 1–2 and the final group included participants with lifestyle score 3–5.

### Outcome ascertainment

Our outcome of interest was all-cause mortality. Mortality information was obtained by the probabilistic linkage of the NHANES III with death certificate records from the National Death Index records conducted by the National Center for Health [32]. The National Death Index has been shown to accurately ascertain participants’ death in a number of studies [33, 34]. Follow-up time was defined as the time (in months) from interview date until death from any cause or end of follow-up (December 31, 2011), whichever came first.

### Statistical analysis

Baseline categorical data were expressed as percentages and continuous data as means and standard errors of the mean (SEM). Cox proportional hazards regression models were used to determine the association between adherence to the lifestyle score or its individual healthy lifestyle behaviors and mortality. Regarding confounder adjustment, the first model (Model 1) was adjusted only for age at study entry (continuous, years), sex, and race/ethnicity (Non-Hispanic white, Non-Hispanic black, Mexican-American, Other). The second model (Model 2) was additionally adjusted for a number of a-priori determined confounders, based on the existing literature, including: time between cancer diagnosis and study entry (continuous, years), socioeconomic status (poor, near poor, middle income, higher income or unknown; categories based on the poverty income ratio, similarly to Suresh et al.[35]), marital status (married/living together, never married/widowed or divorced/separated, similarly to Goldfarb-Rumyantzev et al.[36]), daily energy intake (continuous, kcal/day), and type of cancer diagnosed (female cancers, including breast, ovarian, cervical and uterine cancers, male cancers, including testicular and prostate cancer, gastrointestinal cancers, including esophageal, gallbladder, liver, pancreatic, stomach, colon, rectum, and large intestine cancers, or other cancers). Inclusion of reproductive health information (breastfeeding, parity, age at menarche and menopause) in the full model for female participants did not modify the results and, thus, was not included in the final model. The results were presented as hazard ratios (HR) and corresponding 95% confidence intervals (CI).

To calculate the influence of each of the healthy lifestyle behaviors included in the lifestyle score we estimated the reduction in effect by alternatively excluding each healthy lifestyle behavior from the score and estimating the association with mortality for the remaining ones. To preserve comparability to the full lifestyle score (range 0–5), we multiplied the logarithm of the hazards ratio of the lifestyle scores containing one less behavior (range 0–4) with 5/6 before exponentiation. Then, we calculated the reduction in effect using the following formula (similarly to Trichopoulou et al. [37]): $\frac{\left(1-HR´)-(1-HR\right)}{\left(1-HR\right)}$, where HR´: HR alternatively excluding each healthy lifestyle behavior and HR: HR of the full lifestyle score. To control for possible confounding, whenever a healthy lifestyle behavior was excluded from the lifestyle score we adjusted for it in the fully adjusted model.

All statistical analyses were performed using SAS version 9.4 (SAS Institute, Cary, NC) and significance levels were set at a = 0.05. Sampling weights adapted according to our total study population were used in all analyses to account for the complex survey design and survey non-response.

## Results

Description of the study population and baseline characteristics are shown in Table 1. Mean time between cancer diagnosis and entry in the NHANES III was 9.7 years (SEM: 0.6; for the total study population). The most frequent cancer site was breast, followed by colorectal and prostate cancers. Participants who adhered to none of the healthy lifestyle behaviors entered the study at a slightly younger age and were more likely to be current or former smokers compared to participants who adhered to some or most of the healthy lifestyle behaviors.

**Table 1:** Socioeconomic characteristics and healthy lifestyle behaviors of cancer survivors by lifestyle score category and combined (n = 522)^a^^,^^b^.

	Total Study Population	Lifestyle Score 0	Lifestyle Score 1–2	Lifestyle Score 3–5
N of cancer survivors	522	105	336	81
Age at study entry, years	56.8 (1.1)	54.6 (1.7)	57.4 (1.2)	57.3 (2.5)
Follow-up time, years	14.6 (0.5)	14.1 (0.7)	14.2 (0.5)	16.0 (0.6)
Age at diagnosis, years	47.1 (1.2)	45.5 (2.0)	47.8 (1.4)	46.5 (2.2)
Time between diagnosis and study entry, years	9.7 (0.6)	9.1 (0.9)	9.5 (0.7)	10.8 (0.9)
Sex, %				
Female	72.5	64.2	76.2	69.9
Race/Ethnicity, %				
Non-Hispanic white	88.8	88.2	87.5	93.0
Non-Hispanic black	7.2	9.3	8.1	2.5
Mexican-American	1.9	1.4	2.4	0.9
Other	2.1	1.1	2.0	3.6
Marital status, %				
Married/living together	64.9	66.1	64.3	65.3
Never married/widowed	22.6	15.8	24.5	23.7
Divorced/separated	12.5	18.1	11.2	11.0
Socioeconomic status, %				
Poor	10.2	21.4	8.4	4.2
Near poor	20.3	22.0	22.4	12.7
Middle income	35.5	33.8	35.6	36.8
Higher income	27.6	17.6	25.6	43.8
Missing	6.3	5.2	8.0	2.6
Adherence to individual lifestyle behavior…				
Smoking, %				
Yes	37.3	0.0	43.4	56.8
Physical activity, %				
Yes	34.9	0.0	30.9	82.4
Lifetime healthy body weight maintenance, %				
Yes	28.9	0.0	25.0	69.8
Alcohol consumption, %				
Yes	8.0	0.0	4.7	26.0
High diet quality, %				
Yes	40.3	0.0	43.2	72.4

^**a**^ Age at study entry and age at diagnosis were expressed as mean and standard error of the mean, whereas all remaining variables as percentages.

^**b**^ Adherence in the healthy lifestyle behaviors was defined as: Never smoker, lifetime healthy body weight maintenance (expressed as lifetime highest body mass index 18.5–24.9 kg/m^2^), participation in moderate to vigorous physical activity ≥5 times per week, moderate alcohol consumption (5-15g/day for females and 5-30g/day for males) and high diet quality (expressed as HEI score in the highest 40% of the study population distribution (HEI score > 69.3)).

**Fig 1:**
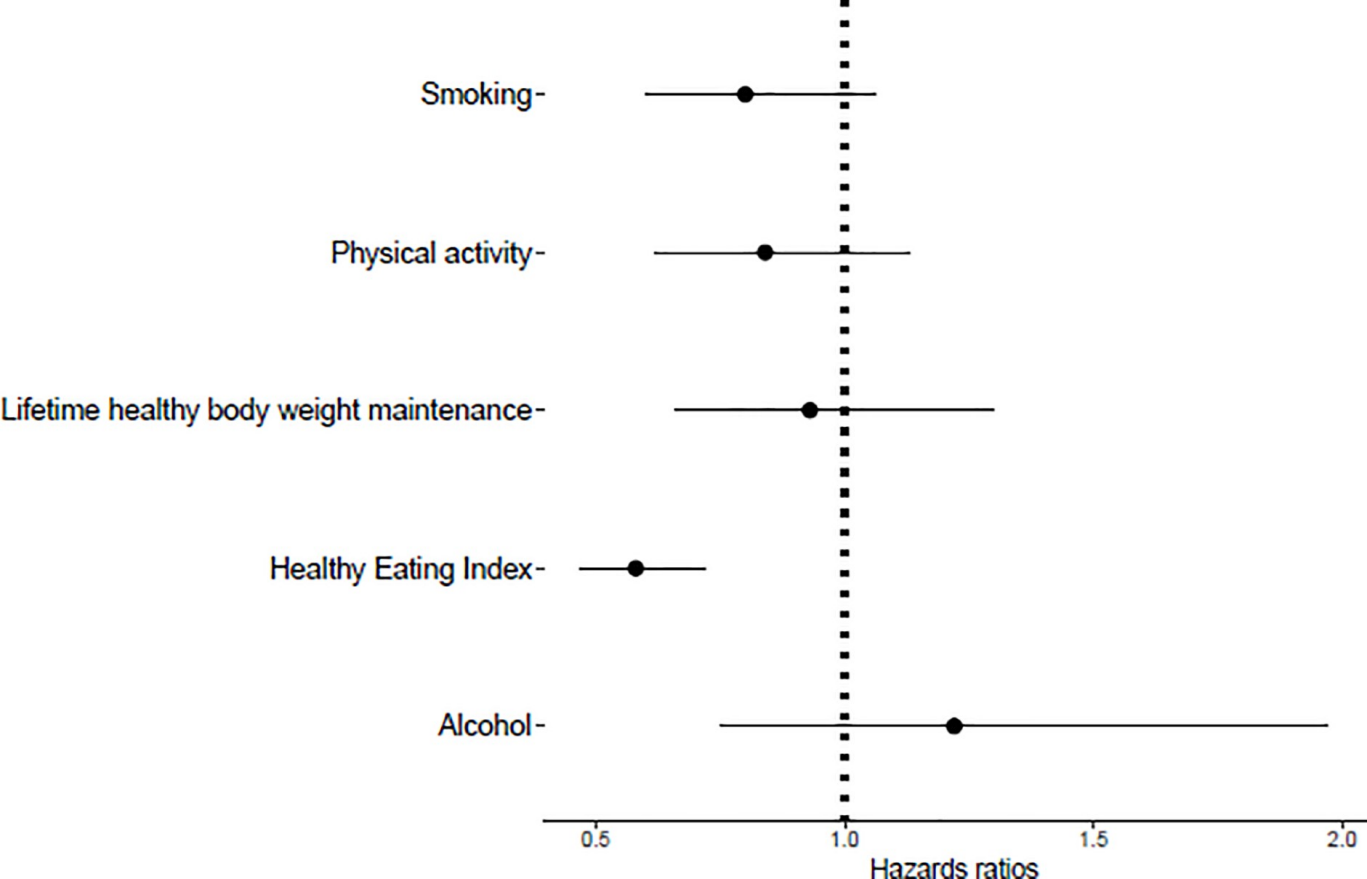
Hazard ratios for adherence to each healthy lifestyle behavior and mortality. Adherence was defined as: Never smoker, lifetime healthy body weight maintenance (expressed as lifetime highest body mass index between 18.5 and 24.9 kg/m^2^), participation in moderate to vigorous physical activity 5 or more times per week, moderate alcohol consumption (5-15g per day for females and 5-30g per day for males) and high diet quality (expressed as HEI score in the highest 40% of the study population distribution (HEI score > 69.3)). Adjusted for: age at study entry (years), sex, race/ethnicity (Non-Hispanic white, Non-Hispanic black, Mexican-American, Other), time between diagnosis and inclusion in the study (years), marital status (married/living together, never married/widowed or divorced/separated), socioeconomic status (poor, near poor, middle income, higher income or unknown), daily energy consumption (kcal/d) and type of cancer diagnosed (female cancers, male cancers, gastrointestinal cancers or other cancers).

The association between adherence to each healthy lifestyle behavior and mortality, investigated after a mean follow-up time of 14.5 years, is shown in Fig 1. The number of deaths recorded until the end of FU was 344 (mean FU time: FU_alive_ = 20.1 years, FU_deceased_ = 9.6 years). Adherence to each healthy lifestyle behavior was inversely associated with mortality in most cases; however, only the adherence to the HEI reached statistical significance (HR_HEI_ = 0.58, 95% CI: 0.47, 0.72). Adherence to moderate alcohol consumption was not associated with mortality (HR_alcohol_ = 1.22, 95% CI: 0.75, 1.97).

The association between the lifestyle score and mortality is shown in Table 2. A 1-unit increase in the lifestyle score was statistically significantly associated with lower mortality in the total population and in sex-specific analyses (HR_total_ = 0.81, 95% CI: 0.72, 0.90; HR_Females_ = 0.79, 95% CI: 0.65, 0.95; HR_Males_ = 0.81, 95% CI: 0.69, 0.95, in the fully adjusted models, per 1 unit increase in the lifestyle score). Compared to null score, moderate (adhering to 1–2 healthy lifestyle behaviors) or high scores (adhering to 3–5 healthy lifestyle behaviors) were statistically significantly associated with mortality risk in the total study population (HR_1-2 vs. 0 total_ = 0.71, 95% CI: 0.56, 0.92; HR_3-5 vs. 0 total_ = 0.57, 95% CI: 0.38, 0.85, in the fully adjusted models). Sex-specific analyses revealed similar results for both sexes, but the association between moderate or higher adherence and mortality failed to reach statistical significance in female participants.

**Table 2:** Hazard ratios for post-diagnostic healthy lifestyle behaviors in association with mortality among cancer survivors by sex and combined (n = 522).

	Total Population (n = 522)				Females (n = 339)				Males (n = 183)			
N of events	344				191				153			
	Model 1^a^		Model 2		Model 1		Model 2		Model 1		Model 2	
	HR	95% CI	HR	95% CI	HR	95% CI	HR	95% CI	HR	95% CI	HR	95% CI
Continuous	0.77	0.69, 0.86	0.81	0.72, 0.90	0.76	0.65, 0.89	0.79	0.65, 0.95	0.79	0.68, 0.92	0.81	0.69, 0.95
Categorical												
Lifestyle Score 0	1.00	Referent	1.00	Referent	1.00	Referent	1.00	Referent	1.00	Referent	1.00	Referent
Lifestyle Score 1–2	0.72	0.56, 0.92	0.71	0.56, 0.92	0.75	0.49, 1.17	0.77	0.46, 1.31	0.65	0.43, 0.98	0.58	0.40, 0.84
Lifestyle Score 3–5	0.50	0.34, 0.71	0.57	0.38, 0.85	0.47	0.28, 0.78	0.54	0.29, 1.01	0.61	0.42, 0.87	0.66	0.45, 0.99

Abbreviations: CI, Confidence interval; HR, Hazard ratio

^a^ Model 1: Adjusted for age at study entry (years), sex and race/ethnicity (Non-Hispanic white, Non-Hispanic black, Mexican-American, Other), Model 2: Additionally adjusted for time between diagnosis and inclusion in the study (years), marital status (married/living together, never married/widowed or divorced/separated), socioeconomic status (poor, near poor, middle income, higher income or unknown), daily energy consumption (kcal/d) and type of cancer diagnosed (female cancers, male cancers, gastrointestinal cancers or other cancers). In sex-specific analyses, sex was not included as a confounder in the model.

The reduction in beneficial effect on mortality with the removal of each healthy lifestyle behavior from the score is shown in Table 3. As expected, the beneficial association between the lifestyle score and mortality attenuated with the alternate removal of each of the healthy lifestyle behaviors. The highest reduction was observed with the removal of the HEI, followed by smoking (58% and 21% reduction in effect, respectively).

**Table 3:** Reduction in effect with the exclusion of each healthy lifestyle behaviors from the lifestyle score.

Healthy Lifestyle Behaviors	Estimate (Logarithmic Scale)	HR	95% CI^a^	Reduction in Effect
Full Lifestyle Score	-0.215	0.81	0.72, 0.90	-
Lifestyle Score Without Healthy Eating Index	-0.087	0.92	0.82, 1.02	57.9%
Lifestyle Score Without Smoking	-0.164	0.85	0.75, 0.96	21.1%
Lifestyle Score Without Lifetime Healthy Body Weight Maintenance	-0.175	0.84	0.76,0.93	15.8%
Lifestyle Score Without Physical Activity	-0.193	0.82	0.75, 0.91	5.3%
Lifestyle Score Without Alcohol	-0.213	0.81	0.73, 0.90	0.8%

Abbreviations: CI, Confidence interval; HR, Hazard ratio

^a^ HR and 95% CI adjusted for age at study entry (years), sex, race/ethnicity (Non-Hispanic white, Non-Hispanic black, Mexican-American, Other), time between diagnosis and inclusion in the study (years), marital status (married/living together, never married/widowed or divorced/separated), socioeconomic status (poor, near poor, middle income, higher income or unknown), daily energy consumption (kcal/d) and type of cancer diagnosed (female cancers, male cancers, gastrointestinal cancers or other cancers). The HR was additionally adjusted for each alternately excluded healthy lifestyle behavior. Before exponentiation the logarithm of the HR was multiplied by 5/6 to preserve comparability between the full lifestyle score and the score excluding one of the healthy lifestyle behaviors.

## Discussion

In our study of cancer survivors, lifetime healthy body weight maintenance, never smoking, regular participation in physical activity, consumption of a high-quality diet and moderate consumption of alcohol, as expressed by a lifestyle score, was associated with lower mortality. Lower mortality was also observed for cancer survivors who only adhered to some of these healthy lifestyle behaviors and the results did not vary significantly by sex.

The inverse association between adherence to a number of healthy lifestyle behaviors and mortality in healthy populations has been reported [31, 38]. The association between combined lifestyle behaviors and the risk of death in cancer survivors has not been investigated extensively, but the existing studies support our findings. Cancer survivors who adhered to healthy weight, physical activity, and diet recommendations, in line with either the American Cancer Society Nutrition and Physical Activity Guidelines [39] or the World Cancer Research Fund / American Institute for Cancer Research Guidelines for Cancer Prevention [40] had lower mortality risk compared to those who did not [4, 24, 25].

Our results further suggest that modifying one to two behaviors could still lead to a lower risk of death, even if a number of lifestyle behaviors are non-modifiable (e.g. ever smoking or lifetime healthy body weight maintenance). This has been supported by studies that have included the adherence to healthy body weight, physical activity, and high-quality diets in their scores and have reported an inverse association [4, 24, 25]. Following only some of the recommendations was also associated with lower risk of premature death in one of the studies [4], suggesting that even partial adherence to healthy lifestyle could be beneficial.

There are several mechanisms by which a healthy lifestyle might influence cancer survivors’ survival. Data suggests that healthy body weight, physical activity and diets high in vegetables, fruits, and whole grains promote insulin sensitivity, decrease inflammation and improve vitamin D levels [41, 42]. These biomarkers have been associated with lower mortality in the literature [41, 43].

Moderate alcohol consumption was included in our lifestyle score and had a positive contribution to the association between healthy lifestyle behaviors and mortality in cancer survivors. The effect of long-term alcohol consumption in cancer survivors has not been studied extensively; however, moderate alcohol consumption has been associated with cardiovascular benefits in cohort studies in healthy populations [44]. Nevertheless, recently a debate has sparked as to whether or not safe levels of alcohol consumption exist [45, 46].

Since cancer survival increases, a number of lifelong health issues pertinent to cancer survivors are emerging. Cancer treatment, genetic predisposition and lifestyle factors may account for the high risk for secondary cancers and other diseases observed in cancer survivors [47]. Adherence to individual healthy lifestyle behaviors or healthy lifestyle guidelines has been associated not only with reduced mortality [4, 10, 13, 15–19, 24, 25] but also with lower prevalence of other health issues [48], better post-treatment physical functioning [49] and better self-reported quality of life in cancer survivors [50–52].

Our study had several strengths. The prospective study design and the follow-up time allowed us to establish a clear period between disease onset, modifiable lifestyle behaviors, and mortality. Additionally, the detailed information collection at baseline allowed for adjusting for various socioeconomic factors that may influence mortality.

However, this study also has a number of limitations. Our results were based on only one measurement of lifestyle behaviors and may not reflect the long-time habits of the population. Dietary intake and alcohol consumption was estimated using 24-hour dietary recall interviews and therefore may not accurately reflect habitual intake. The self-reported highest body weight attained over the life course might have been underestimated by the study participants. However, it was preferred over measured body weight in an effort to minimize possible reverse causation from weight loss due to the cancer diagnosis and/or treatment. Residual confounding by other behaviors (e.g. dietary supplement use) may also be possible. We cannot exclude the possibility of selection bias e.g. cancer survivors that participated in the NHANES III and by extension in our study could have been healthier/more health conscious than cancer survivors who refused to participate in the NHANES III. The number of cancer survivors did not allow us to investigate the association by cancer type. Future studies should aim to further investigate the association in participants with different cancer diagnosis. Finally, since information regarding disease severity or treatment was not available, we were not able to take them into account in our analyses. Yet, given the long time period between cancer diagnosis and the outcome of interest, cancer treatment and severity are unlikely to severely affect our results.

In conclusion, cancer survivors who followed a healthy lifestyle consisting of lifetime healthy body weight maintenance, never smoking, regular physical activity, consuming a high-quality diet, and drinking a moderate amount of alcohol had lower risk of death compared to survivors who did not adhere to these behaviors. Lower mortality was also observed for cancer survivors who only adhered to some of these behaviors and the results did not vary by sex. Additional studies are required in order to verify our findings and to investigate underlying mechanisms of the mortality-adherence association.

Disclaimer: All analyses, interpretations, or conclusions reached are credited to the authors of this manuscript and not to the NCHS, which is responsible only for the initial data.
